# Xylanase treatment of eucalypt kraft pulps: effect of carryover

**DOI:** 10.1007/s00253-024-13027-3

**Published:** 2024-02-15

**Authors:** José M. S. Matos, Dmitry V. Evtuguin, António P. Mendes de Sousa, M. Graça V. S. Carvalho

**Affiliations:** 1 CERES, Department of Chemical Engineering, University of Coimbra, 3030-790 Coimbra, Portugal; 2 CICECO/Department of Chemistry, University of Aveiro, 3810-193 Aveiro, Portugal; 3 Research Institute On Forestry and Paper (RAIZ), Quinta de S. Francisco, 3801-501 Eixo, Portugal

**Keywords:** Xylanase, Biobleaching, Chemical oxygen demand, Eucalypt kraft pulp, Elementary chlorine-free bleaching

## Abstract

**Abstract:**

The influence of pulp carryover on the efficiency of the xylanase (X) treatment of industrial unbleached and oxygen-delignified eucalypt kraft pulps (A1 and A2 pulps, with kappa number (KN) values of 16 and 10, respectively), collected at the same pulp mill, was studied regarding the consumption of bleaching chemicals and pulp bleachability. Another non-oxygen-delignified eucalyptus kraft pulp of KN 13 was received after the extended cooking from a different pulp mill (pulp B). The assays were performed with both lab-washed (carryover-free) and unwashed (carryover-rich) pulps. Both lab-washed and unwashed pulps with carryover were subjected to X treatment, the former being demonstrating considerably higher ClO_2_ savings than the pulps containing carryover. The savings of bleaching reagents were higher when the X stage was applied to the A1 pulp than to the A2 pulp. This advantage of A1 pulp, however, was not confirmed when using unwashed pulps. In contrast, the gains obtained from the X treatment of unwashed pulp A2 were practically as high as those observed for the lab-washed A2 pulp. Furthermore, a similar effect in X stage was recorded for unwashed pulps having close KN: oxygen-delignified A2 pulp and non-oxygen-delignified B pulp. The results suggest that pulp carryover and initial pH were the key factors relating to the effectiveness of X treatment. The application of X treatment to the A2 unwashed pulp (after the oxygen stage) not only saved 20% of the ClO_2_ and 10% of the sodium hydroxide, but also improved the brightness stability of the bleached pulp without affecting its papermaking properties.

**Key points:**

• *Xylanase treatment boosts kraft pulp bleaching*

• *Pulp carryover hinders the xylanase treatment*

• *Nearly 20% of ClO2 and 10% NaOH savings can be reached using xylanase*

## Introduction

Bleached kraft pulp is widely used to produce printing and writing (P&W) papers of high quality. The pulp bleaching is one of the crucial production steps dealing with the removal of concomitant chemical components (mainly lignin) responsible for the brown colour of unbleached kraft pulp (Suess 2010). Bleaching operations implies the use of expensive oxidising reagents (e.g. ClO_2_), which can be also environmentally unfriendly (Sjöström 1993). Although in recent decades the environmental impact of pulp bleaching has progressively decreased, there are still concerns about the production of organochlorine compounds (AOX) (Sixta et al. 2006). Nowadays, elemental chlorine-free (ECF) concept is the most common way of kraft pulp bleaching worldwide (Valchev 2013). This approach is based on the use of chlorine dioxide (ClO_2_) as the main oxidising agent (Bajpai 2012). Although this bleaching agent produces a much lower amount of AOX, comparing to elemental chlorine, Cl_2_ (Valchev 2013; Bajpai 2012; Solomon 1996), the continuous reduction of ClO_2_ consumption for pulp bleaching is required in order to comply with the strict environmental regulations (Suess 2010). Oxygen delignification is a significant alternative step towards further reducing the use of chlorine derivatives in pulp bleaching (Valchev 2013; Bajpai 2012; Colodette and Martino 2013). Oxygen does not produce AOX and is relatively cheap reagent (Sixta et al. 2006; Bajpai 2012), being able to remove 35 to 65% of the residual lignin that is left in pulp after cooking (Sixta et al. 2006). However, the lack of selectivity of oxygen pulp delignification, leading to compromised pulp quality, limits the use of this technology (Colodette and Martino 2013; Bajpai 2012; Sixta et al. 2006; Sjöström 1993). Meanwhile, totally chlorine-free (TCF) bleaching, emerged as an alternative to ECF bleaching to avoid the use of chlorine-based reagents, does not provide the same pulp strength properties required for the P&W paper production (Suess 2010).

In the late 1980s, another approach towards a “greener” bleaching technology emerged dealing with the use of hydrolytic and oxidative enzymes. This enzymatic bleaching technology is based mainly on the use of xylanase, which allowed reducing the consumption of bleaching chemicals, thereby also decreasing AOX load in the wastewater treatment plant (Bajpai 2012; Viikari et al. 1994). Total active chlorine savings in laboratory and mill bleaching trials are generally reported to be between 10 and 25% (Bajpai 2012). Capital investment associated with commercial xylanase technology is relatively low and its implementation at the mill is technically easy, because of its high compatibility with pre-existing industrial facilities. Moreover, xylanase treatment presented itself as an alternative for pulp mills with limited chlorine dioxide production (Foelkel 2013; Bajpai 2012; Suess 2010; Viikari et al. 1994; Buchert et al. 1994). However, there are a few hurdles that may hamper the use of this technology. One of them is the fact that most of the available xylanases present activity in pH and temperature ranges that are incompatible with the conditions they would normally face in the mill (temperature up to 90 °C and highly alkaline pH), meaning increased costs with pulp cooling and acidification (Dhillon et al. 2000). Other problems faced when using xylanase in the pulp mill are not only related to the price of the enzyme, but also to the treatment time and the amount of enzyme to be applied. If the treatment duration and enzyme load are too high, they can lead to excessive xylan hydrolysis and therefore loss of pulp yield, lower beatability of the pulp and loss of fibre strength. However, the development of more thermally robust and efficient enzymes has increased interest in their large-scale applications. Furthermore, advances in enzyme production significantly decreased the market price of enzymes (Lin et al. 2018).

**Fig. 1:**
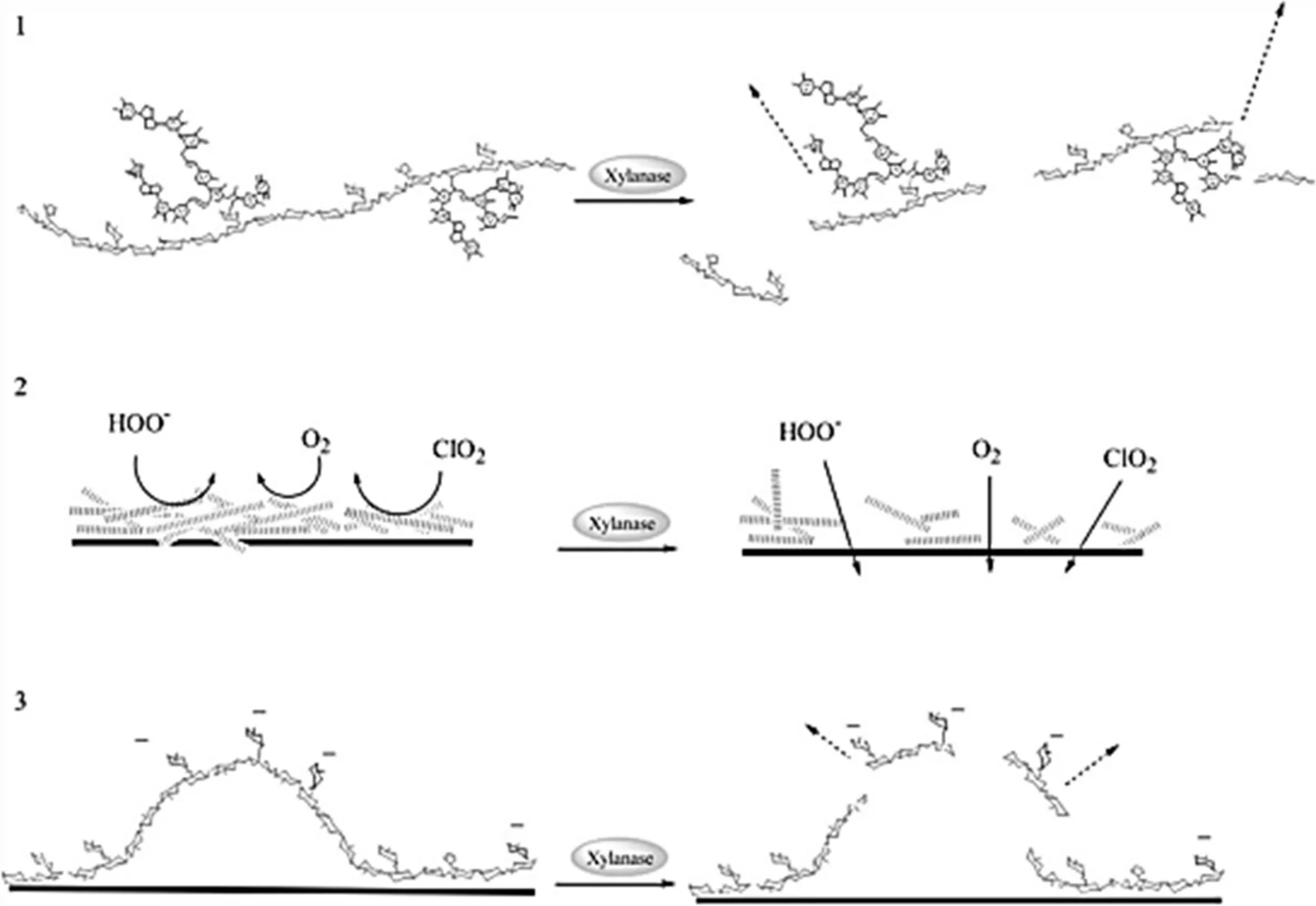
The three main mechanisms of xylanase bleaching: (**1**) release of LCCs; (**2**) removal of redeposited xylans; and (**3**) removal of HexA. Adapted from Henriksson and Teeri (2009)

In pulp bleaching, the term “xylanase” refers commonly to endo-xylanases, which depolymerise xylan (the main hemicellulose in hardwoods) via hydrolysis of glycosidic bonds within its main backbone (Terrasan et al. 2013; Bajpai 2012; Belfaquih et al. 2002). Pulp treatment with xylanase is not a true bleaching stage, as there is no significant delignification happening, but is rather a bleach-boosting step (Sousa et al. 2016; Sixta et al. 2006; Hortling et al. 1994). There are three main proposed mechanisms for the bleach-boosting effect of xylanase (Fig. 1). The first one relates to the cleavage of glycosidic bonds within the xylan backbone that promotes the liberation of lignin fragments linked to the released xylose residues, known as lignin-carbohydrate complexes, or LCCs (de Jong et al. 1997). The second mechanism takes into account that dissolved xylan fragments may re-precipitate on the surface of the fibres towards the end of cooking, and that their removal would mean an increased fibre permeability, thus facilitating the penetration and action of the bleaching agents inside the fibres (Salgueiro et al. 2016; Meng et al. 2015; Roncero et al. 2005; Kantelinen et al. 1993). Lastly, there is the hydrolysis of glycosidic bonds located near xylose residues substituted with hexenuronic acids (HexA) groups, which promotes the release of the latter from pulp. HexA are regarded as chromogenic structures, which contribute mainly to reduce brightness stability in bleached pulp, thus promoting pulp yellowing (Loureiro et al. 2013; Sevastyanova et al. 2006; Granström et al. 2001), and consume bleaching reagents. It is therefore expected that the application of a xylanase treatment to the pulp will promote increased pulp brightness or brightness stability, for the same load of bleaching chemicals, or reduced consumption of chemicals to achieve the same pulp brightness, thus enabling environmental benefits. These gains have been shown to be more significant when enzymatic treatment is applied before bleaching, i.e. as a pre-bleaching treatment (Walia et al. 2017).

The effectiveness of xylanase treatment on kraft pulp is influenced by pulp carryover (Tolan and Foody 1997), which refers to the dissolved organic matter that remains in the pulp slurry after kraft cooking (or after oxygen delignification), and comprises solubilised lignin degradation products and polysaccharides (Matos et al. 2023). The carryover characteristics depend not only on the cooking conditions or oxygen delignification stage, but also on the efficiency of the respective washing operations. The importance of carryover regarding the performance of the enzymatic stage resides in three main aspects: (1) organic compounds deposited on the surface of the fibres (mainly oxidised lignin and hemicelluloses) prevent enzyme penetration into the fibres, and cause enzyme adsorption onto them, thereby impairing its hydrolytic action (Senior et al. 1990); (2) this organic matter is also dissolved in the “liquor” and causes direct enzyme inhibition; (3) if the X treatment requires pH reduction, in order to be compatible with the optimal activity range of xylanase, some of the suspended oxidised lignin may be protonated upon acidification, and re-precipitate onto the surface of the fibres (Zhu et al. 2014; Zhu 2013; Suess 2010; Walia et al. 2017), further impairing xylanase penetration ability and action. For these reasons, both washing efficiency and the location of enzyme addition are expected to play important roles in the success of an enzymatic pulp treatment. Pulp carryover in terms of dissolved organic matter is expected to be much higher after pulping than after oxygen delignification, since most of the lignin dissolved during pulping is removed in subsequent washing operations and much less lignin is released after oxygen delignification. Consequently, it is common practice to apply xylanase treatment after an oxygen delignification step, rather than before it (Tolan and Guenette 1997; Loureiro et al. 2021).

In this work, the viability of applying the xylanase (X) treatment on unbleached eucalypt kraft pulp, compared to the application on oxygen-delignified pulp, was assessed. The main goals were as follows: (i) compare the effect of applying stage X to oxygen-delignified eucalyptus kraft pulp versus direct application to unbleached pulp; (ii) verify whether the application of stage X to unbleached pulp can be considered an effective way to reduce bleaching chemical loads when oxygen delignification has not been implemented; (iii) to analyse the effect of pulp carryover using industrial pulps without additional laboratory washing. Unlike most studies published in this area, this work considered the effect of pulp carryover to accurately evaluate the performance of xylanase treatment. Furthermore, the results obtained in this study can play an important role in understanding whether enzymatic technology could be an efficient solution with regard to saving bleaching chemicals in eucalyptus kraft pulp mills, which do not employ a delignification step with oxygen.

## Materials and methods

### Raw materials

Three industrial eucalypt pulp samples were collected at two distinct Portuguese kraft pulp mills (Table 1). In one of those industrial plants (mill A), an oxygen delignification (O) stage is applied on pulp following the cooking operation, whereas in the other one (mill B), no oxygen treatment is employed and cooking is prolonged. Two pulp samples were collected at mill A, both before the O treatment (unbleached kraft pulp, UKP) and following this stage, prior to the bleaching process (oxygen-delignified kraft pulp, ODKP). At mill B, only unbleached kraft pulp was collected. Following sampling, pulp slurries were centrifuged to approximately 30% consistency, and the centrifugation filtrates were collected. Both centrifuged pulps and their respective filtrates were stored separately at 4 °C before use. A portion of both pulps was extensively washed and stored likewise. A commercially available endo-1,4-β-xylanase product (presumably glycoside hydrolase 10 (GH10) family), Xylio Pre, supplied by Novozymes (Bagsværd, Denmark), was used. The highest bleaching efficiency for the enzymatic solution was found at 90 °C and pH 8, conditions under which it displayed its highest activity value (79,927 U/g).

**Table 1:** Pulp samples collected at mills A and B, and their respective kappa number (KN) and pH values

Pulp sample	Mill	KN	pH
A1—UKP	A	16	11
A2—ODKP		10	9
B—UKP	B	13	9

### Xylanase activity

Xylanase activity determination was based on the hydrolysis of a model xylan substrate (from oat spelts, purchased from Sigma-Aldrich Chem. Comp., St. Louis, MO, USA) by xylanase, which releases xylan fragments in the form of reducing sugars. These were afterwards quantified using the DNS (3,5-dinitrosalicylic acid) colorimetric method. This quantification method is based on the oxidation of carbonyl groups of the reducing sugars to carboxyls, with simultaneous DNS reduction to 3-amino-5-nitrosalicylic acid, which shifts the colour of the solution from yellow to brownish red. Diluted xylanase solution was incubated with xylan substrate solution and Britton-Robinson buffer (pH 8), at 90 °C, for 60 min. Afterwards, DNS solution was added, and the mixture was boiled for 15 min. Samples were then filtered with the help of nylon syringe filters with a 0.45-µm pore diameter, before their absorbance was measured in a Cary 100 UV–Vis spectrophotometer (Agilent Technologies, Santa Clara, CA, USA), at 540-nm wavelength. Xylanase activity is expressed in U/g and is defined as the amount of enzyme which produces 1 µmol of xylose equivalents (sugars with reducing power equivalent to that of xylose), in 1 min. This methodology was adapted from Ghose and Bisaria (1987).

### Xylanase treatment

The xylanase (X) stage was performed at 10% consistency, inside polyethylene bags, placed in a water bath at the desired temperature. In the X treatments of washed pulp, consistency was adjusted with Britton-Robinson (BR) buffer (pH 8), whereas in the studies with unwashed pulp, consistency was adjusted by addition of the filtrate that had been separated from pulp during the initial centrifugation. In the X treatment of washed pulps, pH was adjusted to 8, using BR buffer solution. In the treatment of unwashed pulps, pH was adjusted by addition of sulphuric acid 0.5 M. In the assays without pH adjustment, the pH value during the X stage was that of the industrial pulp sample. Xylanase solution was added to the buffer/filtrate, before addition to pulp. The duration of the X stage was set at 60 min. Table 2 shows the temperature and xylanase load values used in the X stage applied in each of the pulp samples. Control treatments were carried out under the exact same conditions as their respective X treatment, though without enzyme addition. After the treatment, pulp slurries were subjected to vacuum filtration, and the respective filtrates were immediately collected and stored at 4 °C.

**Table 2:** Temperature and xylanase load used in the X treatments applied on each industrial pulp sample (*odp*, oven-dried pulp)

Pulp sample	Temperature (°C)	Xylanase load (g/t odp)
Pulp A1—UKP	90	25, 50
Pulp A2—ODKP	70	
Pulp B—UKP	65	50

### Chemical bleaching sequences

Chemical bleaching consisted of ECF D_0_E_P_D_1_D_2_ sequences employing D and E_P_ stages (D—chlorine dioxide, ClO_2_, stage; E_P_—alkaline extraction with sodium hydroxide, NaOH, reinforced with addition of hydrogen peroxide, H_2_O_2_). Chemical reagent loads, based on oven-dry pulp (odp), as well as temperature and stage duration, were chosen according to the pulp used. Due to the large difference in KN (kappa number) between industrial unbleached (A1) and oxygen-delignified eucalypt (A2) kraft pulps (Table 1), total chemical loads applied during control sequences (cD_0_E_P_D_1_D_2_) varied between 35 and 60 kg/t odp for ClO_2_ (as active Cl_2_), 10 and 18 kg/t odp for NaOH and 2 to 4 kg/t odp for H_2_O_2_. By the end of each stage, a pulp washing step was performed, using ca. 15 times the excess volume of distilled water per weight of oven-dried pulp.

### Pulp characterisation

The bleach-boosting effect of the X stage was assessed in terms of ISO brightness gain, as well as brightness stability (PC Number) improvement. ISO brightness determination was performed according to ISO 2470–1 (2009), using handsheets prepared according to ISO 3688 (1999). Accelerated dry ageing of handsheets was simulated by conditioning them in the dark, at 105 °C, for 60 min. This ageing treatment was followed by another brightness reading, following equilibration to the appropriate temperature (23 ± 1 °C) and relative humidity (50 ± 2%) conditions. ISO brightness was determined using an ABB L&W Elrepho 071 spectrophotometer (Lorentzen & Wettre, Kista, Sweden). PC Number was calculated resorting to Eq. (1) (Liitiä and Tamminen 2007).

1 $$\mathrm{PC\;Number}= 100*\left(\frac{{\left(1-\frac{\mathrm{brightness\;after\;reversion\;}(\mathrm{\%})}{100}\right)}^{2}}{\left(2*\frac{\mathrm{brightness\;after\;reversion\;}(\mathrm{\%})}{100}\right)}-\frac{{\left(1-\frac{\mathrm{initial\;brightness\;}(\mathrm{\%})}{100}\right)}^{2}}{\left(2*\frac{\mathrm{initial\;brightness\;}(\mathrm{\%})}{100}\right)}\right)$$

KN determination was carried out according to ISO 302 (2004). Pulp kappa number (KN) determination relies to oxidation of the oxidisable compounds present in pulp by potassium permanganate (KMnO_4_) under acidic conditions. KN is defined as the volume (in mL) of KMnO_4_ consumed per oven-dried g of pulp, after 10 min of reaction, at 25 °C. KMnO_4_ which is not consumed after that time is immediately determined through reverse iodometric titration.

Determination of pentosan content, hexenuronic acid content and intrinsic viscosity was performed in bleached pulp. Determination of pentosan content consisted of the distillation of furfural produced during hydrolysis of pulp with hydrochloric acid, followed by absorbance analysis, at 630-nm wavelength, in a Cary 100 UV–Vis spectrophotometer (Agilent Technologies, Santa Clara, CA, USA), as described in TAPPI Standard procedure TAPPI T 223 cm-01 (2001). Hexenuronic acid quantification was based on the acid hydrolysis of pulp with sodium formate (pH 3.5), for 6 h, at 105 °C, in nitrogen atmosphere, followed by absorbance analysis of the filtrate of the hydrolysate at 245- and 480-nm wavelengths, in a Cary 100 UV–Vis spectrophotometer (Agilent Technologies, Santa Clara, CA, USA). The used methodology was adapted from the one described by Vuorinen et al. (1999). The method for intrinsic viscosity determination relates to the complete dissolution of the cellulosic chains in a cupriethylenediamine (CED) solution, which does not alter chain length. Viscosity is determined by measuring the flow time of the solution in a viscometer, under standard conditions, as described in ISO 5351 (2010).

### Papermaking properties

Papermaking properties were evaluated on isotropic handsheets of approximately 60 g/m^2^ grammage, produced according to ISO 5269–1 (2005), after refining pulp at 2000 revolutions, in a laboratory refiner (PFI model, L&W MKV, Hamjern, Norway), following ISO 5269–2 (2004). Evaluated pulp properties included as follows: drainability (ISO 5267–1 (1999)), bulk (ISO 534 (2011)), burst index (ISO 2758 (2014)), tensile index (ISO 1924–2 (2008)), tear index (ISO 1974 (2012)), opacity and light scattering coefficient (ISO 2469 (2014)), Klemm capillary rise (ISO 8787 (1986)), Gurley air resistance (ISO 5636–5 (2013)), Bendtsen roughness (ISO 5636–3 2013)) and water retention value (adapted from ISO 23714 (2014)). Handsheet grammage was determined according to ISO 536 (2012).

### Chemical oxygen demand

Chemical oxygen demand (COD) determination relies on the oxidation of almost all organic compounds, either solubilised or suspended in a liquid sample. Filtrate samples were initially oxidised by the digestion with sulphuric acid and potassium dichromate, in the presence of silver sulphate and mercury sulphate (in reaction tubes Hach Lange LCK 514) at 170 °C, for 15 min, in a Hach Lange HT 200 S digital thermostate (Hach Company, Loveland, CO, USA). The amount of dichromate spent in the oxidation of the sample was calculated after absorbance analysis of the formed Cr(III), at 600-nm wavelength, in a Hach Lange DR 2800 spectrophotometer (Hach Company, Loveland, CO, USA). COD measurement was performed according to ISO 15705 (2002).

## Results

### Bleaching of washed pulp

In order to remove all organic compounds that exist, either solubilised in the liquid phase (carryover) of the pulp slurry, and precipitated on the fibre surface, industrial A1 and A2 pulps were thoroughly washed in the laboratory (lab-washed pulps). ClO_2_ savings during bleaching of lab-washed A1 and A2 pulps (D_0_E_P_D_1_D_2_ sequence) were evaluated through a ClO_2_ load reduction during the first D stage (D_0_), or by elimination of the last bleaching stage (D_2_). As the ClO_2_ load added during the D_2_ stage is very low comparing with that applied in the D_0_ and D_1_ stages, D_2_ elimination could not provide as much chemical savings as the first approach. However, it could increase mill productivity and process economy, because of the elimination of an entire bleaching stage. Due to the large difference in KN observed between A1 and A2 pulps (Table 1), the chemical loads applied to each pulp were different, as indicated previously. When the X stage was employed, the ClO_2_ load in the D_0_ stage was reduced by 30% in the bleaching of pulp A1, while a 25% reduction was applied for A2 pulp. The elimination of the last bleaching stage (XD_0_E_P_D_1_) was examined for both pulps.

**Fig. 2:**
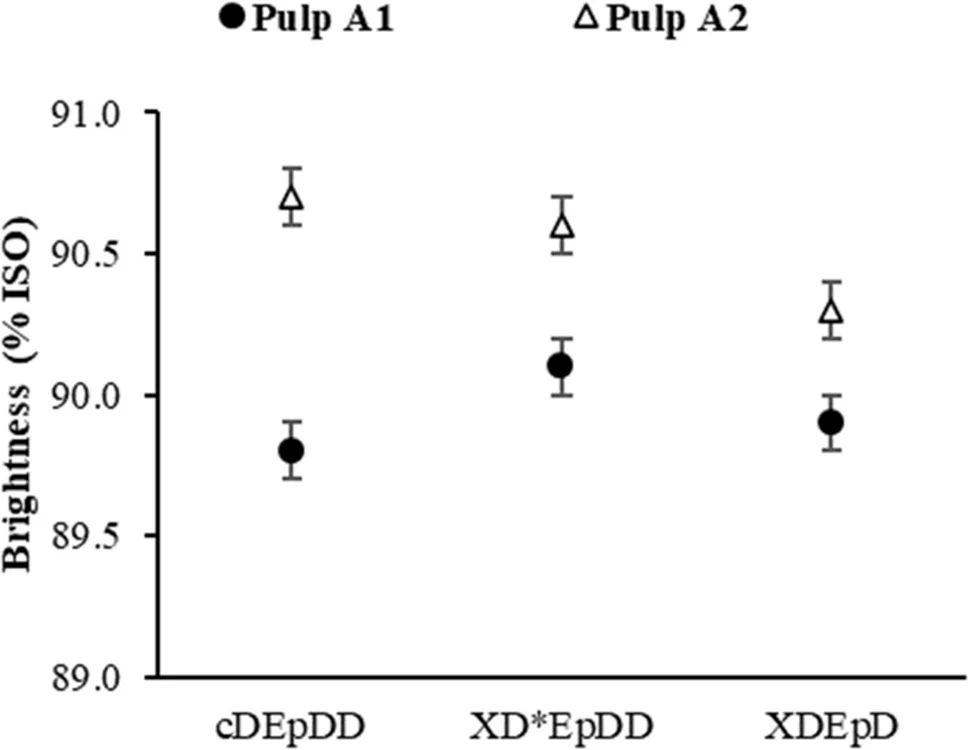
Pulp brightness after bleaching of enzyme-treated and untreated A1 (UKP) and A2 (ODKP) washed pulps. cDE_P_DD—control sequence; XD*E_P_DD—complete sequence incorporating a xylanase treatment (D* refers to modified D_0_ stage—30% ClO_2_ load reduction for pulp A1, and 25% reduction for pulp A2); XDE_P_D—incomplete sequence incorporating a xylanase treatment (D_2_ elimination)

Noteworthy that no significant bleaching gains were detected while increasing the enzymatic load from 25 to 50 g/t odp (data not shown) and further trials were carried out using lowest enzymatic charge (25 g/t odp). The obtained data suggest that the X treatment on pulp A1 allowed the decrease of the ClO_2_ load in D_0_ by 30%, or, alternatively, the elimination of the D_2_ stage, without decreasing brightness of the bleached pulp (Fig. 2). On the other hand, the reduction of ClO_2_ in D_0_ by 25%, or the elimination of D_2_, after the enzymatic treatment of A2 pulp, impairs insignificantly the brightness development. As in the bleaching of UKP (A1 pulp), higher ClO_2_ loads were applied (60 kg/t odp), because of its higher KN value (ca. 16), the ClO_2_ savings (absolute values) achieved with this pulp in X treatment were also considerably higher compared to the ODKP pulp (A2 pulp), which total ClO_2_ load was 35 kg/t odp only. Looking at two options to lessen a load of ClO_2_ in D_0_ or in D_2_ stage, it seemed that for both lab-washed A1 or A2 pulps, the former choice (ClO_2_ reduction in D_0_) was preferable, being in tune with results reported in other works on enzymatic bleaching of hardwood kraft pulp (Ko et al. 2010). The findings of the present study also agree with the results by Kantelinen et al. (1993), who reported the xylanase treatment to be less effective for the pulps with lower KN. It is also important to note that the thermal stability of the employed xylanase was not compromised while increasing the X treatment temperature from 70 °C for A2 pulp to 90 °C for A1 pulp.

### Bleaching of unwashed pulp in short bleaching sequence

In view of the expected impaired effectiveness of enzyme treatment by both carryover and pulp pH, the effect of X stage on unwashed pulps (sampled at the mill, without undergoing a laboratory washing procedure) was evaluated using a short bleaching trial DE_P_ (E_p_ stage after the first D_0_ step). Presumably, after the E_P_ stage, the eventual benefits of X stage introduction before the bleaching should be visible.

**Fig. 3:**
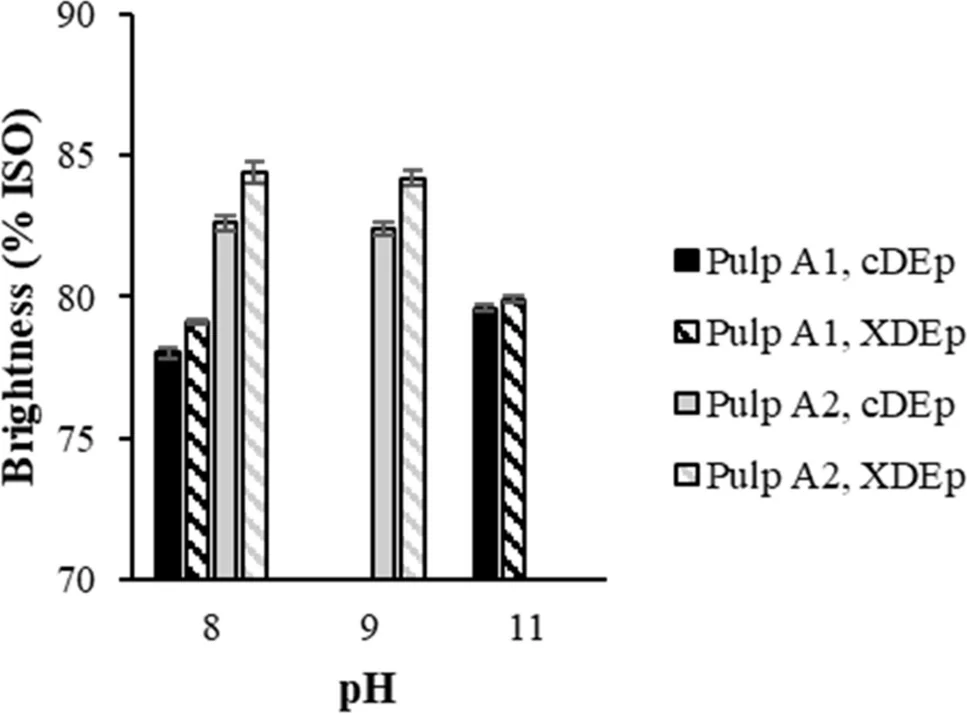
Pulp brightness development in short bleaching sequences involving enzyme-treated and untreated unwashed A1 (UKP) and A2 (ODKP) pulps. cDE_P_—control sequence without enzyme; XDE_P_—sequence including enzymatic treatment (X)

The results on X stage implementation on unwashed pulp without pH adjustment (pH 11 in the case of A1 pulp and pH 9 for A2 pulp) were compared with those with pH adjustment as used in the study with washed pulps (pH 8) (Fig. 3). The development of pulp brightness with the pH decrease in X stage revealed a negative effect, especially for the A1 pulp. For this pulp, decreasing the pH from 11 to 8 in the control treatment (cDE_P_) led to a 1.6% ISO brightness decrease after E_P_. On the other hand, the enzymatic treatment for the same A1 pulp seemed more efficient in terms of brightness gains when applied at pH 8, as it presented a brightness increase of 1.1% ISO, when compared to the control assay without enzymatic treatment (vs. 0.3% ISO difference at pH 11). This is explained by the fact that the applied xylanase shows the highest efficiency at pH close to 8 (see the “Materials and methods” part). Nonetheless, although X stage was most effective at pH 8 (Fig. 3), the acidification to that pH value affects negatively the ISO brightness of A1 pulp reached in DE_P_ bleaching (79.1%ISO), which is even lower than that obtained without pH adjustment and enzyme addition (79.6%ISO). Thus, the best pulp brightness was obtained when the X stage was applied at a pH value farther from the optimum for the enzyme. However, it is not necessary that the pH reduction had an adverse effect on the enzymatic activity, because the leachable carryover components (dissolved low molecular weight residual lignin and polysaccharide degradation products) could precipitate on the pulp surface at pH below 10, thus affecting its brightness (Colodette and Martino 2013; Lisboa et al. 2005). In contrast, the lack of effectiveness in X stage at pH 11 (A1 pulp) (Fig. 3) can be explained by the excessively high pH value, which was outside the ideal range of xylanase activity (nearly pH 8).

Changes in the KN of pulp and COD of the filtrate, after both control treatments of pulp A1, were also evaluated (Table 3). When pH of A1 pulp suspension was not adjusted in the control treatment simulating X stage, the outcoming pulp possessed a KN value approximately 1 unit lower than the initial A1 pulp. However, when the same control treatment was carried out at pH 8, the treated pulp did not show any decrease in KN (Table 3). Moreover, whereas the COD value of the filtrate originated from the non-adjusted control treatment at pH 11 was 16.6 g/L (1.6 g/L increase comparing to the COD of the initial liquor), adjustment to pH 8 caused COD to decrease (0.7 g/L) to 14.3 g/L. These results confirmed the fact that a significant amount of soluble organic matter in the carryover was leached from the pulp at pH 11, but was not removed at pH 8 and retained on fibres. This agrees with previous suggestions about importance of carryover matter on the pulp bleachability at different pH.

**Table 3:** Effect of pH adjustment before the control treatments of unwashed pulp A1 (unbleached kraft pulp) on pulp kappa number (KN) and on the chemical oxygen demand (COD) of the filtrates from the treatments

	Pulp KN (units)	COD of the filtrate (g/L)
Pulp A1	16	15.0
Control treatment at pH 11 (pulp A1)	15	16.6
Control treatment at pH 8 (pulp A1)	16	14.3
Pulp A2	10	2.7

### Chlorine dioxide savings in the fully bleached unwashed A2 pulp

Since only A2 pulp from mill A was shown to be the most suitable for the application of the xylanase pre-bleaching treatment on unwashed pulp, this pulp was involved in the D_0_E_P_D_1_D_2_ full bleaching trials. The enzymatic stage was carried out at pH 9; thus, no pH adjustment was necessary. The 20% ClO_2_ in D_0_ and 10% NaOH in E_P_ reductions were adjusted. The possibility of elimination of D_2_ stage was also evaluated.

Noteworthy the practically unaltered pulp brightness and significantly improved brightness stability (expressed as the PC Number) of the A2 pulp bleached using XD_0_E_P_D_1_D_2_ sequence operated with reduced loads of bleaching reagents (Fig. 5). Comparing to XD_0_E_P_D_1_D_2_ bleaching, the shorter bleaching sequence XD_0_E_P_D_1_ produced pulp with lower final brightness (90.4 vs. 89.6%ISO) and brightness stability (PC Number of 0.19). These results confirm that the last D_2_ stage, although employing a relatively low ClO_2_ load (ca. 10% from the total), is still very important regarding brightness development and stability.

### Pulp characterisation following full bleaching of unwashed pulp A2

Xylan removal during the X treatment is to be expected since this hemicellulose is the direct target of xylanase attack. Hemicelluloses are known to play an important role in improving pulp refining and papermaking properties. Accordingly, pentosan content in the enzyme-treated bleached pulp was compared to the control pulp treated without xylanase addition. Eucalypt hemicelluloses are almost exclusively xylans (Lisboa et al. 2005); therefore, xylan removal from pulp should be visible in the form of a decrease in pentosan content. Residues of hexenuronic acid (HexA), which exist as substituents derived from glucuronic acid attached to the xylan backbone (Loureiro et al. 2013; Roncero et al. 2005), were also quantified. As expected, a drop in pentosan content (*ca.* 1% odp) was visible in the pulp that was pre-treated with xylanase (Fig. 6).

The papermaking properties after implementation of X pre-treatment stage (XD_0_E_P_D_1_D_2_) were compared with those of conventionally bleached unwashed A2 pulp (D_0_E_P_D_1_D_2_) (Table 4). Basic strength and optical properties were evaluated for unbeaten pulps.

**Table 4:** Papermaking properties of fully bleached pulp following complete bleaching of enzyme-treated and control unwashed oxygen-delignified kraft pulp (ODKP, pulp A2)

	cD_0_E_P_D_1_D_2_	XD_0_E_P_D_1_D_2_
Drainability (°SR)	28.0 ± 0.0	28.0 ± 0.0
Bulk (cm^3^/g)	1.32 ± 0.00	1.34 ± 0.01
Burst index (kPa m^2^/g)	5.15 ± 0.16	5.00 ± 0.19
Tensile index (kN m/kg)	68.8 ± 3.6	71.3 ± 2.5
Tear index (mN m^2^/g)	9.28 ± 0.52	9.16 ± 0.48
Light scattering coefficient (m^2^/kg)	33.4 ± 0.7	33.8 ± 0.6
Opacity (%)	69.1 ± 0.5	69.0 ± 0.4
Klemm capillary rise (mm)	4.3 ± 0.1	4.4 ± 0.2
Gurley air resistance (s/100 mL)	8.5 ± 0.5	8.4 ± 0.4
Bendtsen roughness (mL/min)	85.5 ± 3.9	89.0 ± 4.4
Water retention value (%)	133 ± 4	134 ± 1

## Discussion

Regarding the results obtained on the lab-washed A1 and A2 pulps (Fig. 2), similar features in ClO_2_ savings were found in previous studies regarding ECF bleaching sequences, achieved after xylanase treatment of either unbleached or oxygen-delignified eucalypt kraft pulp. Thus, in one of those studies, the treatment of eucalypt kraft pulp with a purified bacterial xylanase, at 60 °C and pH 7, for 3 h, in a XDED bleaching sequence, achieved savings in ClO_2_ consumption of up to 30% (Kiddinamoorthy et al. 2008). In another study, the treatment of unbleached mixed eucalypt (*Eucalyptus grandis* and *Eucalyptus urophylla*) kraft pulp with a recombinant commercial xylanase, at 50 °C and pH 6.5, for 1 h, before a DE_P_D bleaching sequence (XDE_P_D) was employed, where the xylanase pre-treatment resulted in a 36% reduction in total active chlorine consumption (Meng et al. 2015). Vidal et al. (1997) assessed the xylanase treatment of unbleached *E. globulus* kraft pulp, at 45 °C, and pH 7–8, for 3 h, which resulted in 34% total ClO_2_ consumption decrease in a XODPD bleaching sequence. Elsewhere, oxygen-delignified *E. globulus* kraft pulp was treated with a bacterial xylanase, at 45 °C and pH 7, for 2 h, in a XDE_P_D bleaching sequence; a reduction of over 30% ClO_2_ consumption was achieved (Torres et al. 2000). Similarly to results from other studies, enzymatic pretreatment of a pulp with a higher KN (A1 pulp) showed better bleaching with greater ClO_2_ savings than a pulp with a lower KN (A2 pulp) (Fig. 2). However, compared to the aforementioned works, the present study was carried out under more realistic from an industrial point of view treatment conditions (temperature of 70 to 90 °C, pH 8 and treatment duration of 60 min) and using a commercial enzyme product.

Unlike enzymatic treatments of laboratory-washed A1 and A2 pulps, the study of unwashed A2 pulp in the short DE_P_ bleaching sequence showed that substantially higher brightness values were achieved after E_P_ stage, compared to A1 pulp (Fig. 3). Moreover, decreasing pH from the original value (pH 9) to ca. 8 did not affect brightness development in the control treatment, nor the efficacy of the enzymatic stage (1.8% ISO gain). The explanation is the carryover effect. Comparing to A1 pulp, the impact of carryover in A2 pulp was clearly less significant, as seen by the low COD of the filtrate (2.7 g/L in A2 pulp vs. 15.0 g/L in A1 pulp, Table 3). Previously, very satisfactory results with the same xylanase were obtained at pH 9 during the processing of eucalyptus pulps pre-delignified with oxygen (Matos et al. 2023). The lower organic carryover observed for this pulp results from the effect of the oxygen delignification stage, which removes the major part of the organic compounds that remain in solution after kraft cooking, thereby significantly lowering COD. This means that the interference of organic compounds on enzyme activity is largely minimised. In fact, other researchers have previously demonstrated that higher COD values impair X stage efficacy (Fillat et al. 2010). At the same time, insignificant xylanase inhibition by pulp carryover has been reported by other researchers (Curado 2018). Furthermore, as the pH of the A2 pulp carryover is considerably lower (pH 9), its reduction to pH 8 will not be as impactful as when it was reduced from pH 11 to pH 8 for A1 pulp. In fact, data of Fig. 3 indicate that the adjustment of pH in unwashed A2 pulp is unnecessary for the xylanase treatment, because a final brightness was approximately the same at both pH values and no apparent deterioration of xylanase activity at pH 9 took place.

According to the results obtained, it can be suggested that the carryover has a negative impact on the xylanase treatment of eucalyptus kraft pulp, especially at high levels of COD and with a decrease in pH. The most deteriorating effect may be associated with the re-precipitation of dissolved lignin and other pulp degradation products from carryover onto the pulp surface, which impairs the action of xylanase. The effect of carryover on proper xylanase activity should be quite moderate. This study also showed that the conclusions gained in experiments with ideal lab-washed pulps under idealised conditions (e.g. pH and temperature) are not necessarily the same as those obtained with real pulps containing carryover under conditions close to industrial ones. Thus, although the X treatment at 70–90 °C and pH 8 allowed greater ClO_2_ savings in the bleaching of lab-washed pulp A1 compared to pulp A2 (Fig. 2), its application on pulp A1 under realistic mill conditions (pH 11 and 70 °C) did not show any benefits (Fig. 3). In contrast, in the latter case, X treatment of A2 pulp seems to be a viable option to decrease the consumption of bleaching chemicals at mill A operating with an oxygen delignification stage. The lack of sufficient washing of the A1 industrial pulp to lower COD levels and pH of the respective carryover does not allow the successful application of the enzymatic treatment (X stage) in the applied ECF bleaching sequence.

**Fig. 4:**
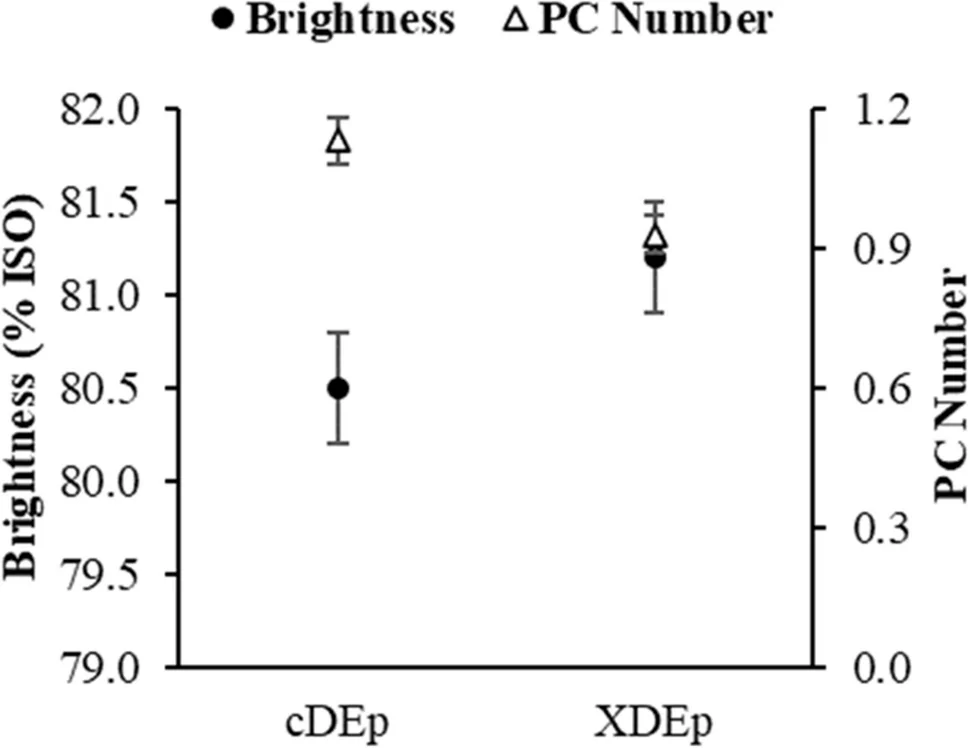
Pulp brightness and brightness stability (PC Number) following short bleaching sequences of enzyme-treated and control unwashed unbleached kraft pulp from mill B (pulp B). cDE_P_—control sequence; XDE_P_—sequence incorporating a xylanase treatment

In order to confirm the importance of pulp washing on the efficacy of the X treatment, industrial pulp B was also examined (Fig. 4). This pulp was collected from a mill in which an extended cooking process is used without oxygen delignification, i.e. kraft cooking is followed by the first D stage in DE_P_DED bleaching sequence. The collected unbleached B pulp possessed a lower KN (13 vs. 16) and pH of a carryover (9 vs. 11) when comparing to pulp A1. Thus, the COD of B pulp was also less than half that of A1 pulp. This unwashed B pulp was subjected to an incomplete XD_0_E_P_ bleaching sequence. A control sequence (cD_0_E_P_) was also performed for comparative reasons (Fig. 4). In the XD_0_E_P_ sequence, the loads of ClO_2_, NaOH and H_2_O_2_ were reduced by 10, 10 and 20%, respectively, when compared to parent D_0_E_P_ sequence. Application of X stage allowed superior pulp brightness and brightness stability compared to the control sequence without X stage, providing the lower loadings of bleaching chemicals (Fig. 4). In contrast to A1 pulp from mill A (Fig. 3), the clearly observed bleaching enhancing effect of treatment X employed on this unbleached B pulp (Fig. 4) provides further proof of the importance of pulp washing efficiency in its response to xylanase treatment. Considering these results, it can be assumed that the xylanase bleaching technology might be an interesting tool to be used on unbleached pulp by mills, which do not employ oxygen-delignification, but cook the pulp to quite low KN and have efficient washing operations after pulping. Otherwise, this technology is apparently more effective if applied after the O stage, taking into account the decrease in COD and pH levels of the respective pulp carryover. To the best of our knowledge, the impact of pulp washing operations on xylanase treatment performance has only previously been reported by Tolan and Foody (1997).

**Fig. 5:**
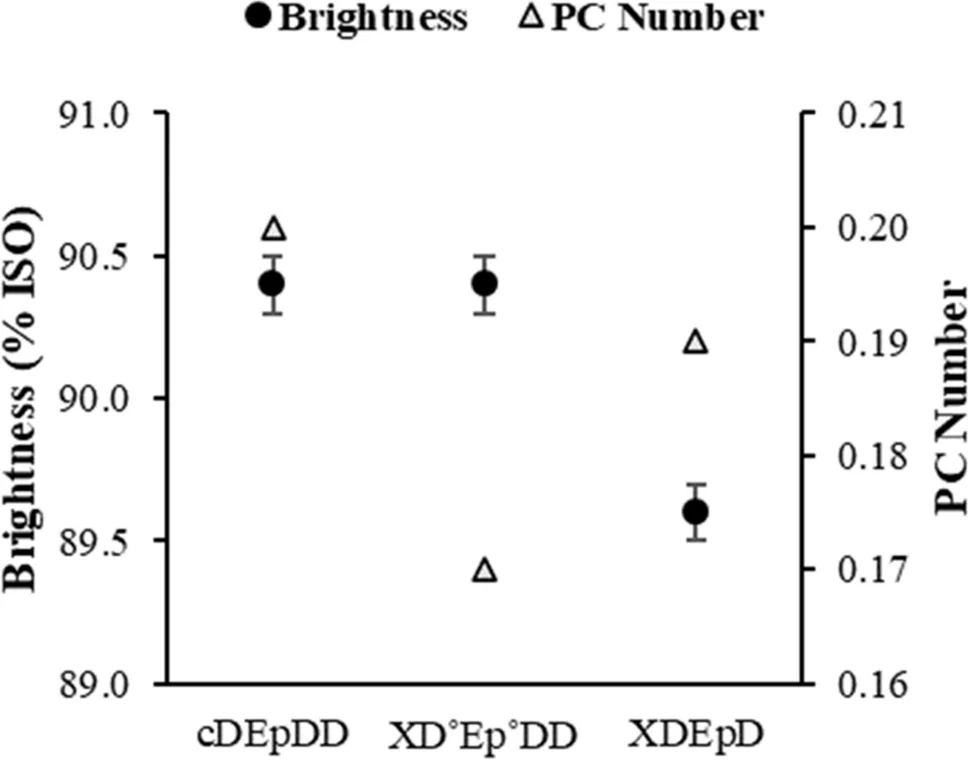
Pulp brightness and brightness stability (expressed as PC Number) of fully bleached enzyme-treated and control unwashed oxygen-delignified kraft pulp (ODKP—A2 pulp). cDE_P_DD—control sequence without X stage; XDE_P_DD—full bleaching sequence with enzymatic (X) treatment (D^o^ and E_P_^o^ refer to modified D_0_ and E_P_ stages, 20% ClO_2_ load reduction in D_0_ and 10% NaOH load reduction in E_P_); XDE_P_D—incomplete bleaching sequence with eliminated D_2_ stage

The results on the implementation of the X treatment prior the full ECF bleaching sequence (XD_0_E_P_D_1_D_2_) of unwashed A2 pulp not only allowed to decrease the ClO_2_ load in D_0_ stage by 20%, and the NaOH load in E_P_ by 10%, but also improved the optical properties of the bleached pulp (brightness stability) (Fig. 5). The last feature is confirmed by reduction in PC number in xylanase treatment that is commonly explained by the removal of chromophore structures/chromogens in structurally associated residual lignin and partially degraded xylan (Gangwar et al. 2016, 2015). At the same time, elimination of D_2_ stage with simultaneous application of pre-bleaching X stage impermissibly impairs the brightness of fully bleached pulp and is impractical (Fig. 5).

**Fig. 6:**
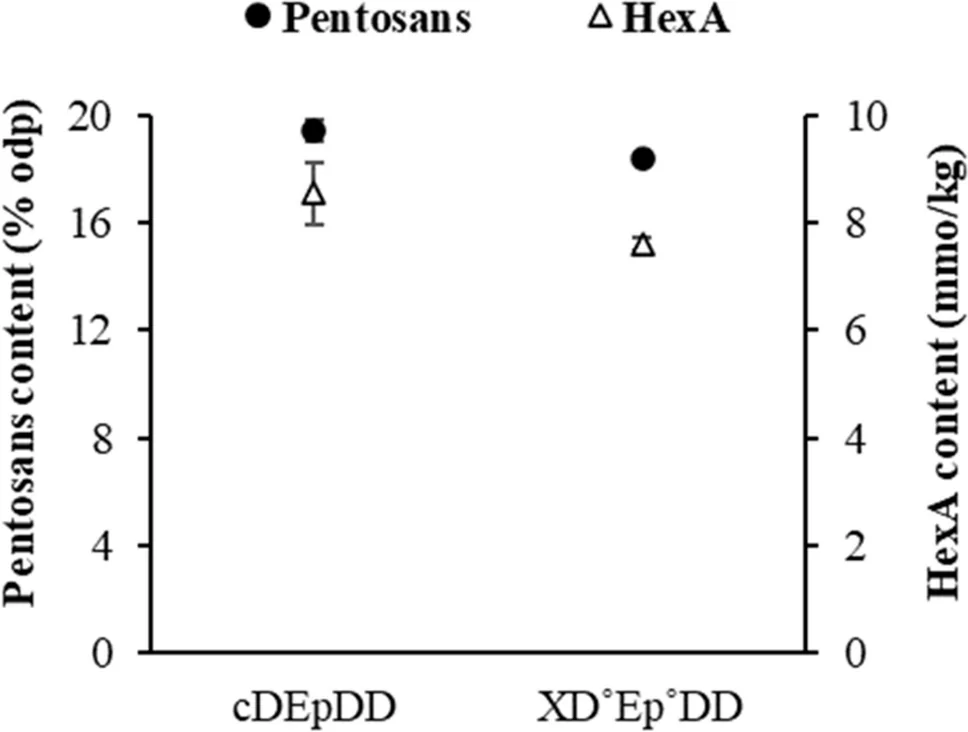
Pentosan and hexenuronic acid (HexA) contents in bleached pulps obtained in enzyme-treated and control trials of unwashed oxygen-delignified kraft pulp (ODKP—A2 pulp). cDE_P_DD—control sequence without X stage; XDE_P_DD—full bleaching sequence with enzymatic (X) treatment (D^o^ and E_P_^o^ refer to modified D_0_ and E_P_ stages, 20% ClO_2_ load reduction in D_0_ and 10% NaOH load reduction in E_P_)

The removal of xylan, determined as pentosans, in X stage of the full XD_0_E_P_D_1_D_2_ bleaching of A2 pulp (Fig. 6) was consistent with removal of HexA. Pentosan content reduction after xylanase treatment has also been reported by Angayarkanni et al. (2006). A slight reduction in HexA content was also observed, probably as a result of the removal of xylan fragments containing these groups (Valls et al. 2010). The decrease in HexA content by xylanase was also previously reported by other researchers (Meng et al. 2015; Borges et al. 2013; Fillat et al. 2012; Gallardo et al. 2010). Knowing that HexA is readily degraded by ClO_2_ (Törngren and Ragnar 2002), the fact of its further reduction in the fully bleached pulp by the implementation of stage X presumes its indirect removal by the action of xylanase outweighed the effect of the decrease in the ClO_2_ load (Fig. 6). As HexA are chromogenic structures, their decrease in enzyme-promoted bleaching explains the improvement in pulp brightness reversal (lower PC number). Similar features have also been reported by Borges et al (2013).

The release of xylan from the pulp resulted in an increase in COD in the effluent from stage X (4.2.g/L), compared to the filtrate from the control treatment of A2 pulp (3.4 g/L) (Table 3). This difference is probably due to the dissolution of xylan fragments released from pulp (Vidal et al. 1997; Roncero et al. 1996). Regarding the degradation of cellulose in pulp during X treatment, the intrinsic viscosity obtained for control pulp was 946 ± 9 dm^3^/kg, while for enzyme-treated pulp, a value of 932 ± 5 dm^3^/kg was detected. This difference is not significant, meaning that the X stage in general did not affect the cellulose counterpart of pulp, which indicates the absence of cellulase activity in the xylanase sample, which otherwise could also degrade the cellulose in the pulp (Foelkel 2013; Kumar et al. 2016; Beg et al. 2001; Walia et al. 2017). The lack of xylanase effect on pulp viscosity is indeed often reported (Terrasan et al. 2013; Valls et al. 2010; Betini et al. 2009; Torres et al. 2000; Bim and Franco 2000; Máximo et al. 1998), being an indicator of relative purity in relation to the presence of other hydrolytic enzymes.

Savings in chemicals are unimportant unless pulp quality requirements are met, namely concerning its papermaking qualities. Hemicelluloses, such as xylan, provide free hydroxyl groups on the fibre surface, which promote the formation of hydrogen bonds between fibres, thereby strengthening inter-fibre bonding (Meng et al. 2015), and resulting in better physical properties of the fibre web (Gangwar et al. 2014). Xylan molecules also facilitate refining, by increasing fibre swelling and reducing the hornification effect, also due to the presence of accompanying carboxylic groups from 4-*O*Me-α-D-glucuronic and HexA moieties (Sousa et al. 2017). As a result of the removal of hemicelluloses by xylanase, decreased papermaking quality of pulp previously treated with xylanase, namely lower drainability (Fillat et al. 2012) and strength properties, is often reported (Meng et al. 2015; Borges et al. 2013). As xylanase treatment affects xylan removal essentially from the fibre surface (Gangwar et al. 2014; Meng et al. 2015), this may explain the negligible changes in drainability of enzymatically treated and untreated pulps (Table 4). It has previously been suggested that the refinability of eucalyptus kraft pulps is mainly affected by xylan, which occurs in bulk of the cell wall rather than on the fibre surface (Sousa et al. 2017). The apparently unchanged fibre structure after enzymatic treatment may explain that in the examined trials, no significant impact was observed on any of the analysed strength (burst, tensile and tear indexes), optical (light scattering coefficient and opacity) and structural (capillary rise, air resistance, roughness and water retention value) paper properties on fully bleached pulps (Table 4). Similar observations have been made by other authors regarding the xylanase effect in kraft pulp bleaching (de Araújo et al. 1999; Qu et al. 1996). However, a weak tendency towards an increase in the bulk and tensile index of pulps fully bleached with X treatment was observed. These observations confirm that partial removal of xylans from eucalyptus kraft pulp (estimated to be around 1% of the pulp dry weight) did not cause the decrease in its basic papermaking properties.

The results of this study evidenced bleaching boosting effect gained with the application xylanase treatment stage (X stage) using an industrial thermotolerant enzyme in unbleached and oxygen delignified eucalyptus kraft pulps. The most significant bleaching results were obtained when enzyme treatments were performed at the beginning (e.g. in XDE_P_DD or OXDE_P_DD) rather than at the end (e.g. in DE_P_DX) of the ECF bleaching sequences. The effect of the xylanase treatment depends strongly on the pulp sampling point in a kraft pulp line, which is also related to the respective carryover composition. Thus, pulp cooked with a high kappa number (KN) without a pre-delignification step with oxygen is the least suitable for enzymatic treatment due to the large amount of dissolved organic matter (COD) in the concomitant pulp carryover making X treatment inefficient. In contrast, delignification with oxygen or extended cooking to relatively low KN favours the effect of the X treatment due to the relatively low COD of carryover and the pH that most closely approximating the ideal (pH 8–9). The negative effect of kraft pulp carryover in the enzymatic treatment is mainly associated with the denial of xylanase access to the surface of the pulp fibre, due to the precipitation of dissolved lignin and polysaccharide degradation products, and not with the decrease in xylanase activity in the bulk solution. Nearly 20% of ClO_2_ and 10% NaOH savings can be reached in ECF bleaching (OXDE_P_DD or XDE_P_DD) with minimal pulp yield loss (about 1%) without deterioration of pulp papermaking properties. It has also been suggested that the results of laboratory studies obtained on the xylanase treatment of lab-washed idealised kraft pulps cannot be directly extrapolated to actual industrial pulps under real ECF bleaching conditions without taking into account the concomitant carryover effect.

## Data Availability

The datasets generated during and/or analysed during the current study are available from the corresponding author on reasonable request.
